# Immunomodulation to Prevent or Treat Neonatal Sepsis: Past, Present, and Future

**DOI:** 10.3389/fped.2018.00199

**Published:** 2018-07-19

**Authors:** Simone S. Schüller, Boris W. Kramer, Eduardo Villamor, Andreas Spittler, Angelika Berger, Ofer Levy

**Affiliations:** ^1^Division of Neonatology, Pediatric Intensive Care & Neuropediatrics, Department of Pediatrics and Adolescent Medicine, Medical University of Vienna, Vienna, Austria; ^2^Precision Vaccines Program, Division of Infectious Diseases, Department of Medicine, Boston Children's Hospital, Boston, MA, United States; ^3^Harvard Medical School, Boston, MA, United States; ^4^Department of Pediatrics, Maastricht University Medical Centre (MUMC+), Maastricht, Netherlands; ^5^School for Oncology and Developmental Biology (GROW), Maastricht University, Maastricht, Netherlands; ^6^Department of Surgery, Research Labs & Core Facility Flow Cytometry, Medical University of Vienna, Vienna, Austria; ^7^Broad Institute of MIT and Harvard, Boston, MA, United States

**Keywords:** neonatal sepsis, preterm infant, adjuvant sepsis therapy, immunomodulation, pentoxifylline, lactoferrin, probiotics, human milk

## Abstract

Despite continued advances in neonatal medicine, sepsis remains a leading cause of death worldwide in neonatal intensive care units. The clinical presentation of sepsis in neonates varies markedly from that in older children and adults, and distinct acute inflammatory responses results in age-specific inflammatory and protective immune response to infection. This review first provides an overview of the neonatal immune system, then covers current mainstream, and experimental preventive and adjuvant therapies in neonatal sepsis. We also discuss how the distinct physiology of the perinatal period shapes early life immune responses and review strategies to reduce neonatal sepsis-related morbidity and mortality. A summary of studies that characterize immune ontogeny and neonatal sepsis is presented, followed by discussion of clinical trials assessing interventions such as breast milk, lactoferrin, probiotics, and pentoxifylline. Finally, we critically appraise future treatment options such as stem cell therapy, other antimicrobial protein and peptides, and targeting of pattern recognition receptors in an effort to prevent and/or treat sepsis in this highly vulnerable neonatal population.

## Introduction

Despite advances in neonatal care leading to improved survival rates and reduced complications in preterm infants (1), there has been little improvement in the prophylaxis, treatment, and adverse neurodevelopmental outcomes associated with neonatal sepsis over the last three decades (2–5). The incidence of neonatal sepsis is inversely correlated with gestational age (GA) and birth weight (BW). While ~20% of very low BW (<1,500 g; VLBW) infants suffer from one or more systemic infections during their hospital stay (6, 7), the rate may reach up to 60% in the most immature infants (8). Inflammatory conditions such as neonatal sepsis and necrotizing enterocolitis (NEC) are associated with persistently high morbidity and mortality rates in these infants (9, 10).

## Definition and clinical course of neonatal sepsis

While a consensus definition of pediatric sepsis exists, defined as a systemic inflammatory response syndrome (SIRS) in the presence of suspected or proven infection (11), no such consensus definition has yet been published for neonatal sepsis (12). Although a positive blood culture defines bacteremia and has been included in some proposed definitions of neonatal sepsis, such an approach does not take into account that in most cases of neonatal sepsis clinical signs are not associated with positive blood cultures (13). Recent studies included a combination of laboratory tests, clinical findings and/or the duration of antimicrobial treatment (≥5 days), reflecting complexity and heterogeneity in neonatal sepsis (3). Thus, in clinical practice, diagnosis of neonatal sepsis is complicated by the absence of a consensus definition, non-specific symptoms, and low sensitivity of the low volume bacterial blood cultures typically obtained (12). Furthermore, established diagnostic tests to predict severity and to guide treatment are lacking (3). Complete blood counts, including immature to total neutrophil ratios, C-reactive protein (CRP), interleukin (IL)-6 or CXCL-8 (IL-8), and procalcitonin (PCT) have some clinical utility (14). Cell surface markers on circulating cells, such as soluble CD14, CD64, and HLA-DR, offer some diagnostic value (15–17). However, much remains to be learned regarding optimal diagnostics and research in this area, including the application of systems biology approaches for biomarker discovery (18).

Early empiric treatment with antibiotics is essential for neonatal bacterial sepsis. Rapid clinical deterioration, however, may still ensue even if antibiotic treatment is started promptly. Possible life-threatening complications include the development of disseminated intra-vascular coagulation, pulmonary hypertension, congestive heart failure and shock (19). These complications can result from phases of excessive inflammation as well as immunosuppression (20–22). Until recently, the immunological basis of sepsis was thought to be a biphasic process with an initial hyperinflammatory phase followed by a later anti-inflammatory phase manifesting as functional immune suppression (23). However, genome-wide transcription profiling in human sepsis of term neonates, children, and adults demonstrates that phases of pro- and anti-inflammatory mechanisms occur during variable times over a sepsis episode and that patients may cycle through each phase multiple times during the course of sepsis (22, 24–28).

## Immunological risk factors for neonatal sepsis

Little is known regarding the sepsis phases in preterm human neonates, but recent findings indicate that both gestational and postnatal age are significant factors affecting immune responses during the critical window of immune adaptation (20, 29, 30). During this window, pathogen-associated molecular patterns (PAMPs) and damage-associated molecular patterns (DAMPs) are potent inducers of inflammation and might shape immune responses early in life (31). Stimulation of pattern recognition receptors (PRRs) in human preterm blood by exogenous PAMPs induces T helper (Th) and anti-inflammatory profiles with impaired Th1 and pro-inflammatory cytokines (32). In this context, innate immunity in newborns has often been alluded to as “impaired,” “defective,” or “immature.” However, it is more accurately described as “distinct” since the fetus/preterm neonate is well-equipped for life *in utero* and the term newborn immune system appropriately mediates the transition from *in utero* to *ex utero* life. For maintenance of tolerance to maternal antigens and to avoid inflammation-triggered preterm delivery, neonatal immune responses are in general T helper (Th)-2 and Th-17 cell biased (33). However, such polarization also corresponds to GA-dependent susceptibility to invasive infections (32, 34). In addition, decreased complement-mediated/phagocytic activity (35, 36), reduced absolute neutrophil counts and functions (37), as well as altered phenotype and function of professional antigen-presenting cells (APCs) (38–40) in response to most Toll-like-receptor agonists (TLRAs) have been described. Moreover, the distinct composition of neonatal plasma, including high concentrations of adenosine (41–44), prostaglandins (45), placental hormones such as cortisol (46), estradiol, and progesterone (47), inhibits the production of Th1 cytokines. In addition, adenosine may also contribute to impaired neutrophil responses by inhibiting neutrophil-endothelial adhesion molecules (48). In contrast, high perinatal levels of cytokines such as the migration inhibitory factor (MIF) (49) and d-dopachrome tautomerase (DDT, also known as MIF-2) (50) counter regulate the activity of adenosine and prostaglandin E2 and together with interleukin-18 (51) further shape immune responses early in life. Taken together, a tightly regulated distinct balance of pro- and anti-inflammatory mediators in neonates shapes early life innate immune responses.

Of note, with respect to human *in vitro* assays, whereas responses to most pure TLRAs are reduced in early life, live organisms such as Group B *streptococcus* (GBS) may signal robust inflammatory responses including TNF production (52). GBS signals in part via TLR8 (53), a TLR pathway that recognizes microbial viability (54). GBS in human newborn blood potentially acts via vita-PAMPs, that is a subset of PAMPs expressed specifically by live microorganism (55), and TLR8 that detects bacterial RNA (52, 54, 56) and might therefore contribute to an exaggerated inflammation in bacterial neonatal sepsis *in vivo* (20). In addition, pro-inflammatory IL-1β responses are impaired in cord blood of preterm infants, but are restored to adult levels during the neonatal period, indicating rapid maturation of these responses after birth (57). Systemic inflammation characterized by high concentrations of proinflammatory cytokines are associated with poor long-term outcomes, and preterm infants are especially vulnerable to inflammatory injuries (10).

A non-inflammatory profile with mature immunoregulatory capacities is acquired in an age—dependent manner and a delayed adjustment of regulatory signaling pathways might in part explain the preterm infants' susceptibility to inflammatory conditions. Neonatal cord blood of term neonates contains extremely high concentrations of alarmins that act as endogenous DAMPs (58). *In utero*, alarmins induce an “endotoxin-like state” by altering MyD88-dependent pro-inflammatory gene programs with corresponding low Th1 responses (30). After birth, alarmin-levels decrease and regulatory TIR-domain-containing adapter-inducing interferon-β (TRIF)-dependent genes gradually increase (30, 58). A disruption of this critical sequence of transient alarmin programming and subsequent reprogramming of regulatory pathways, as occurs in preterm birth, increase the risk of hyperinflammation and neonatal sepsis (30). Of note, alarmins are increased in intra-amniotic infection/inflammation (59) and histologic chorioamnionitis correlates with a decreased risk of LOS (60) indicating that perinatal inflammation may enhance the immunoregulatory and/or functional maturation of the preterm immune system.

Other counter-inflammatory mechanisms, such as increased numbers of regulatory T cells (61, 62), and myeloid-derived suppressor cells (63, 64), as well as impaired phagocytosis-induced cell death (65, 66) might further affect the outcome of neonatal sepsis. However, the role of these mechanisms in neonatal sepsis has not yet been fully delineated, mostly because of limited access to neonatal peripheral blood samples and difficulties in performing longitudinal studies to investigate neonatal immune ontogeny.

## Epidemiological risk factors of neonatal sepsis

In addition to GA-specific immunological characteristics, sex (67), genetic predisposition (68, 69), the evolving microbiome/ microbial colonization (70), and underlying medical conditions shape immune responses and impact the risk of developing neonatal sepsis (4, 71). Given the number of cellular and molecular factors involved, emerging systems biology offer new avenues to monitor functional immune development in the near future (72–74).

The high susceptibility of preterm infants to invasive infections and associated poor long-term outcomes, have prompted the exploration of alternative therapies for neonatal sepsis. A number of interventions have been evaluated, and some such as oral lactoferrin have demonstrated promise (75), but beyond antibiotics and supportive care, there is presently no approved drug for the treatment or prevention of sepsis in preterm or term neonates (25, 76). This review summarizes past and present immunomodulatory concepts and outlines novel potential targets for the prevention and treatment of neonatal sepsis. A literature search for published meta-analyses, randomized controlled trials (RCTs), systematic reviews, individual clinical studies and emerging work from animal models was performed. A list of current approaches discussed in this article is presented in Table 1 (treatment) and Table 2 (prevention); the corresponding literature search strategy is outlined in Supplementary Figure 1.

**Table 1:** Meta-analyses on adjunctive therapy for neonatal sepsis.

**Intervention**	**Population**	**Outcome**	**RR (95% CI)**	**RCTs/infants**	**References**
Pentoxifylline	All infants with confirmed or suspected sepsis	All-cause mortality to discharge	**0.57 (0.35**–**0.93)**	6/416	(77)
	All Infants with confirmed sepsis	All-cause mortality to discharge	**0.37 (0.19**–**0.73)**	4/235	(77)
	Preterm infants with confirmed or suspected sepsis	All-cause mortality to discharge	**0.38 (0.20**–**0.71)**	4/277	(77)
IVIG (polyvalent or IgM-enriched)	All infants with suspected infection	All-cause mortality to discharge	0.95 (0.80–1.13)	9/2527	(78)
IVIG (IgM-enriched)	All infants with suspected infection	All-cause mortality to discharge	0.68 (0.39–1.20)	4/267	(78)
GM-CSF or G-CSF	All infants with confirmed or suspected sepsis	All-cause mortality to 14 days	0.71 (0.38–1.33)	7/257	(79)
	All infants with confirmed or suspected sepsis	All-cause mortality to discharge	0.53 (0.25–1.16)	5/178	(79)
	Neutropenic infants with confirmed or suspected sepsis	All-cause mortality to discharge	0.38 (0.16–0.95)	3/97	(79)
Granulocyte transfusion	Neutropenic infants with confirmed or suspected sepsis	All-cause mortality to discharge	0.89 (0.43–1.86)	3/44	(80)
	Neutropenic preterm infants with confirmed or suspected sepsis	All-cause mortality to discharge	0.94 (0.39–2.24)	2/33	(80)

*Cochrane reviews or the most updated meta-analysis on the topic were selected for inclusion in the table. Outcomes were selected based on relevance. Statistically significant results are marked in bold. CI, confidence interval; GM-CSF, granulocyte-macrophage colony stimulating factor; G-CSF, granulocyte colony stimulating factor; IVIG, intravenous immunoglobulin; RCT, randomized controlled trial; RR, risk ratio*.

**Table 2:** Meta-analyses on preventive strategies for sepsis in preterm infants.

**Intervention**	**Outcome**	**RR (95% CI)**	**RCTs/infants**	**References**
IVIG	All-cause mortality	0.89 (0.75–1.05)	15/4125	(81)
	Mortality (infectious)	0.83 (0.56–1.22)	10/1690	(81)
	Late-onset sepsis	**0.85 (0.74**–**0.98)**	10/3795	(81)
INH-A21	All-cause mortality	0.80 (0.59–1.08)	2/2488	(82)
	Staphylococcal infection	1.07 (0.94–1.22)	2/2488	(82)
Altastaph	All-cause mortality	1.31 (0.30–5.70)	1/206	(82)
	Staphylococcal infection	0.86 (0.32–2.28)	1/206	(82)
Pagibaximab	All-cause mortality	1.16 (0.82–1.64)^a^	2/1669	(83, 84)
	Staphylococcal infection	1.17 (0.90–1.50)^a^	2/1669	(83, 84)
GM-CSF	All-cause mortality	1.05 (0.64–1.72)	4/639	(79, 85)
	Late-onset sepsis	1.05 (0.84–1.30)	3/564	(79, 85)
Donor human milk vs. formula	All-cause mortality	0.75 (0.44–1.27)	4/721	(86)
	Invasive infection	0.89 (0.67–1.19)	2/219	(86)
	Necrotizing enterocolitis	**0.36 (0.18**–**0.71)**	6/431	(86)
Probiotics (single or multiple strains)	All-cause mortality	**0.77 (0.65**–**0.92)**	27/8056	(87)
	Late-onset sepsis	**0.86 (0.78**–**0.94)**	37/9416	(88)
	Invasive fungal infection	**0.48 (0.33**–**0.71)**	6/916	(89)
Probiotics (single strains)	All-cause mortality	0.95 (0.72–1.26)	11/3424	(90)
	Late-onset sepsis	**0.86 (0.76**–**0.97)**	14/3455	(88)
Probiotics (multiple strains)	All-cause mortality	**0.67 (0.50**–**0.89)**	10/2867	(90)
	Late-onset sepsis	**0.85 (0.74**–**0.97)**	23/5691	(88)
Oral lactoferrin	All-cause mortality	0.65 (0.37–1.11)	6/1041	(75)
	Late-onset sepsis	**0.59 (0.40**–**0.87)**	6/886	(75)
Oral lactoferrin + probiotics	All-cause mortality	0.54 (0.25–1.18)	1/496	(75)
	Late-onset sepsis	**0.27 (0.12**–**0.60)**	1/319	(75)
Glutamine	All-cause mortality	0.97 (0.80–1.17)	12/2877	(91)
	Late-onset sepsis	0.94 (0.86–1.04)	11/2815	(91)
Selenium supplementation	All-cause mortality	0.92 (0.48–1.75)	2/549	(92)
	Late-onset sepsis	**0.73 (0.57**–**0.93)**	3/583	(92)

*Cochrane reviews or the most updated meta-analysis on the topic were selected for inclusion in the table. Outcomes were selected based on relevance. Statistically significant results are marked in bold. Altastaph, antibody against capsular polysaccharide antigen type 5 and 8; CI, confidence interval; GM-CSF, granulocyte-macrophage colony stimulating factor; INH-A21, pooled generic antistaphylococcal immunoglobulin; IVIG, intravenous immunoglobulin; Pagibaximab, anti-lipoteichoic acid monoclonal antibody; RCT, randomized controlled trial; RR, risk ratio*.

a *No complete meta-analysis has been conducted. RR, calculated from preliminary results*.

## Prior studies that have not demonstrated clinical benefit

### Immunoglobulins

Preterm neonates born prior to 32 weeks gestation have low levels of passively acquired antibodies, and endogenous immunoglobulin (Ig) synthesis does not begin until 24 weeks of life (93, 94). Immunoglobulins provide opsonic activity, activate complement, and promote antibody-dependent cytotoxicity (95). Given these biological effects and the observation of decreased Ig levels in severe sepsis, several clinical studies have investigated the use of intravenous immunoglobulins (IVIG) to prevent and treat neonatal sepsis (78, 96–98).

A 2015 Cochrane Review, evaluating 9 IVIG studies and including a total of 3,973 infants showed no reduction of in-hospital mortality or in the combined outcome of death or major disability at 2 years of age in preterm infants with suspected or proven infection (Table 1) (78). Additionally, IgM-enriched IVIG preparations, which may provide higher opsonization activity and complement activation compared to IgG, did not significantly reduce mortality during hospital stay in infants with suspected sepsis (*n* = 66) (78). Based on these findings, routine administration of IVIG or IgM-enriched IVIG to prevent mortality in infants with suspected or proven neonatal infection cannot be recommended.

Given that >50% of cases of late-onset sepsis (LOS) in VLBW are caused by *Staphylococcus* spp., various type-specific antibodies targeted at different antigenic markers of *Staphylococcus* have been developed and studied in RCTs (82, 83). A 2009 Cochrane Review evaluated the effects of two anti-staphylococcal immunoglobulins, INH-A21 (pooled generic anti-staphylococcal immunoglobulin) and Altastaph (human polyclonal immunoglobulin against capsular polysaccharide antigens type 5 and 8), on the prevention of LOS in infants ≤ 32 weeks (82). No significant reduction in the risk of infection or mortality was identified (Table 2). A third anti-staphylococcal immunoglobulin, pagibaximab (anti-lipoteichoic acid monoclonal antibody) was studied, but again no significant reduction in sepsis or mortality was found (83, 84)(Table 2).

The reasons for these failed trials have not yet been elucidated but one might speculate that distinct immune responses in the preterm infant such as reduced complement or leukocyte activity may play a role or that the correct type or combination of antibodies has not yet been found (83). Future studies need to identify antibodies that (i) target optimal epitopes, (ii) have optimal bioactivity and/or (iii) can be targeted to carefully defined populations that are most likely to benefit from them. Studies of protective neutralizing antibodies against neonatal pathogens are ongoing (99). Prior to human studies, such investigations should evaluate the activity of potential immunomodulating products in an age-specific manner including in age-specific human *in vitro* platforms as well as in preterm animal models.

### Glutamine

Endogenous glutamine, a conditionally essential amino acid, is insufficiently biosynthesized in states of metabolic stress. Accordingly the supplementation with glutamine improved clinical outcomes in critically ill adults (100). Glutamine is abundant in human milk, but levels in formula are much lower and it is not routinely supplemented in parenteral nutrition solutions for neonates.

Despite its potential role in metabolic stress, a systematic review of 12 RCTs including 2,877 VLBW did not find any effect of preventive glutamine supplementation on mortality or major neonatal morbidities (Table 1) (91). It remains unknown, whether glutamine supplementation may be beneficial in the recovery of critically ill infants, in particular, after gastrointestinal inflammatory processes such as NEC.

### Antioxidants: selenium, melatonin, and vitamin A

Preterm neonates are at increased risk of oxidative stress due to lower basal levels of plasma antioxidants and metal-binding proteins (ceruloplasmin, transferrin), reduced activity of antioxidant enzymes, and higher potential for exposure to reactive oxygen species (101–103). A 2003 Cochrane review including 297 preterm neonates (<32 weeks of gestation and/or BW ≤ 2,000 g) showed a significant reduction of sepsis episodes associated with prophylactic selenium supplementation but no difference in survival (Table 2) (92). A more recent RCT confirmed these findings (104); an updated meta-analysis is pending. Thus, selenium supplementation in preterm infants might reduce the incidence of sepsis, but does not affect overall mortality.

Melatonin has antioxidant, anti-inflammatory and anti-apoptotic properties that may improve neonatal sepsis outcome, in particular in mitochondrial injury (105). Three small single-center studies investigated the use of melatonin as adjuvant therapy in neonatal sepsis and results indicate a beneficial effect of melatonin (106–108). However, so far no follow-up RCTs have been conducted, thus, firm conclusions here are precluded.

No reduction of neonatal sepsis in vitamin A-treated patients has been demonstrated so far (109), and a recent meta-analysis could not demonstrate a significant reduction of mortality in term neonates, who were supplemented with vitamin A (110). Further results of on-going clinical trials investigating the effect of vitamin A for the treatment of sepsis and NEC are still pending (111).

### Granulocyte and granulocyte-macrophage colony stimulating factors

Myeloid colony stimulating factors (CSFs), including granulocyte-macrophage CSF (GM-CSF; CSF-2) and granulocyte CSF (G-CSF; CSF-3), stimulate innate immune function, improve myelopoiesis, and limit apoptosis. (112). The clearance of apoptotic cells is essential for the resolution of inflammation and phagocytosis of apoptotic granulocytes is diminished in neonates compared to adults (113). Neonates rapidly deplete their small neutrophil pool when septic, resulting in neutropenia and Gram negative sepsis was partially reversed by administration of G-CSF in preterm infants (114, 115). Thus, a number of clinical trials investigated the effect of G-CSF and GM-SCF in the prevention and treatment of neonatal sepsis over the last decades. A 2003 Cochrane Review of seven treatment and three prophylaxis studies however, demonstrated no significant survival advantage at 14 days from the start of therapy (79) (Table 1). Of note, a subgroup analysis of 97 infants, who in addition to systemic infection, had clinically significant neutropenia at trial entry, did show a significant reduction in mortality (79). Three prophylactic studies, on the other hand, did not demonstrate significantly reduced mortality in neonates receiving GM-CSF (79). The authors concluded that due to the small sample size, there was insufficient evidence to support the introduction of either G-CSF or GM-CSF into neonatal practice, either as treatment of established systemic infection to reduce resulting mortality, or as prophylaxis to prevent systemic infection in high-risk neonates (79).

A study from 2009 investigating 280 neonates ≤ 31 weeks' gestation demonstrated that even higher doses of postnatal prophylactic GM-CSF (10 μg/kg per day administrated subcutaneously on 5 consecutive days) did not reduce sepsis or improve survival or short-term outcomes (85). When this study was pooled with the three previously published small RCTs, no significant effects of prophylactic GM-CSF on mortality or sepsis incidence were observed (79, 85) (Table 2).

In summary, available data do not support the use of G- or GM-CSF for prophylaxis of infections in neonates. This might in part be explained by a hyporesponsiveness of neonatal granulocytes to G- or GM-CSF induced anti-apoptotic effects compared to adults (116). Preterm neonates with moderate (<1,700/μL) or severe (<500/μL) neutropenia and systemic infection, however, might benefit from adjuvant treatment with G-CSF or GM-CSF, respectively (117). Optimal timing of administration and monitoring of G- or GM-CSF levels will be crucial to maximize the beneficial aspects of these cytokines in these infants.

### Granulocyte transfusions

Granulocytes of term and preterm neonates exhibit quantitative and qualitative differences compared to those of adults, which may contribute to the neonates' higher risk for developing bacterial infections (114). Treatment of neonatal sepsis with granulocyte transfusions was thus investigated to determine whether it might enhance quality and quantity of neutrophils thereby leading to improved outcome. However, no significant difference in mortality was found in infants with sepsis and neutropenia who received granulocyte transfusions when compared to placebo (Table 1) (80). Of importance, potentially severe side effects have been reported: fluid overload, transmission of blood-borne infection, graft-vs.-host disease, pulmonary complications secondary to leukocyte aggregation and sequestration and sensitization to donor erythrocyte and leukocytes (80, 101). Thus, the application of granulocyte transfusions cannot be recommended due to insufficient evidence of safety and efficacy in preterm infants.

### Exchange transfusions

Exchange transfusion may remove toxic bacterial products and potentially harmful circulating inflammatory mediators, including cytokines, in an effort to improve perfusion and tissue oxygenation, replace clotting factors, and enhance humoral immune responses.

One retrospective and one prospective single center study investigated the effect of exchange transfusions in a cohort of 101 and 83 preterm infants, respectively. In these cohorts of preterm infants with severe sepsis/septic shock no significant reduction in mortality rates was found (118, 119). Nevertheless, a trend in mortality reduction was reported (119) and another study found a statistically significant protective effect, after controlling for potential confounding factors significantly associated with death (GA, serum lactate, inotropic drugs, oligo/anuria) (118). Although hypoglycemia, electrolyte disturbances, hemodynamic instability, and thrombosis are potential complications of exchange transfusion, the authors reported no side effects (120). Thus, based on the current evidence and safety data, no firm conclusion on the recommendation of exchange transfusions for the treatment of neonatal sepsis can be made.

### Recombinant activated human protein C

Recombinant human activated protein C (rhAPC) possesses a broad spectrum of activity including modulation of coagulation, inflammation, and apoptosis (121). However, results among adults and children demonstrated lack of efficacy and an increased risk of bleeding associated with higher mortality rates (122, 123). Consequently, rhAPC was withdrawn from the market before any randomized trials were performed in preterm neonates, and in 2012 a clear recommendation against the treatment with rhAPC for neonatal sepsis was proclaimed (124).

## Current interventions with clinical evidence of benefit

### Human milk

**Figure 1:**
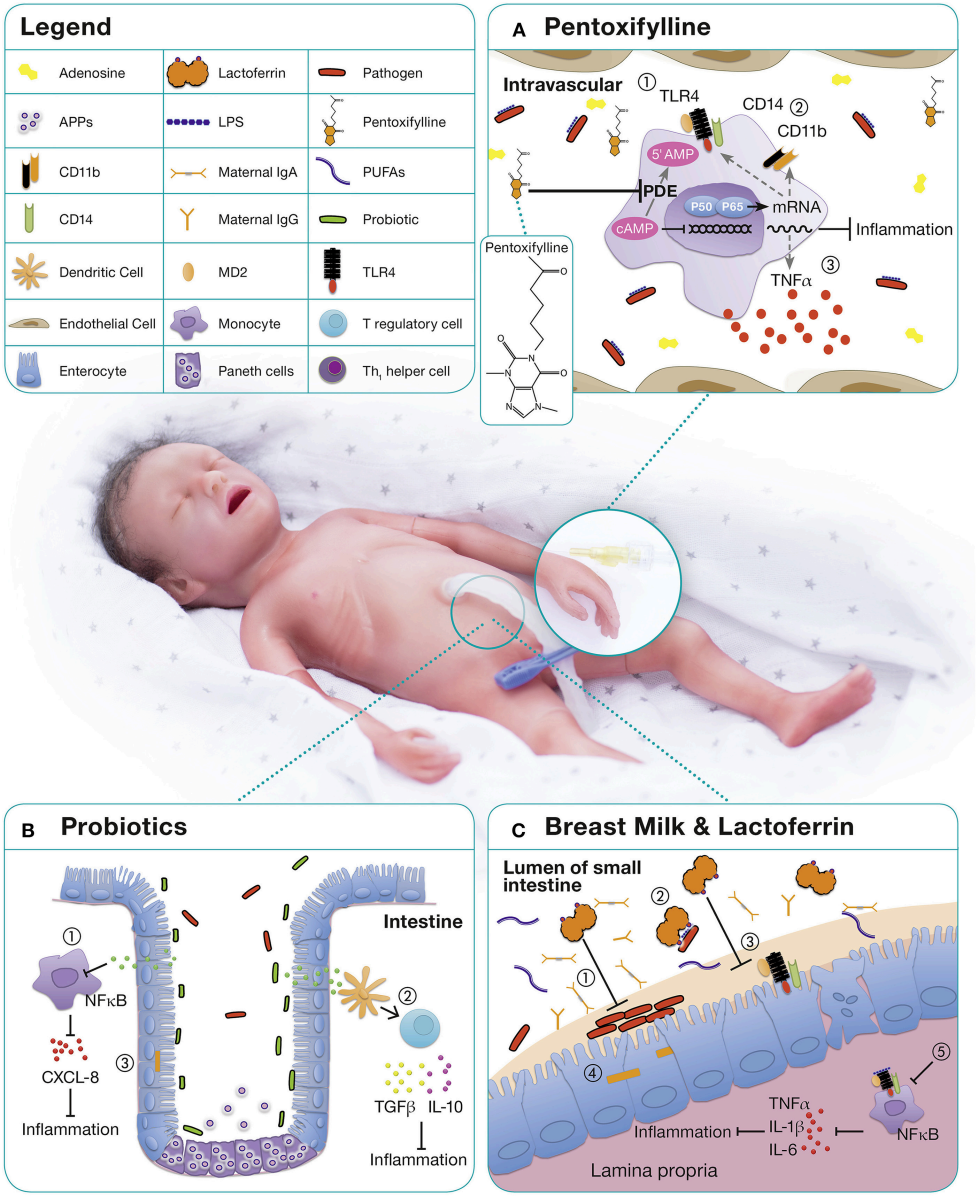
Immunomodulatory approaches for the treatment and prevention of neonatal sepsis. **(A)** PTX, a phosphodiesterase inhibitor, mediates most of its functions by enhanced cyclic AMP (cAMP) due to a reduced degradation of cAMP (125, 126). Relatively high concentrations of adenosine are present in neonatal blood plasma and neonatal mononuclear cells demonstrate increased sensitivity to the cAMP-mediated inhibitory effects of adenosine (127, 128). As immunomodulatory properties of PTX are mediated via adenosine-dependent pathways, adenosine and PTX in combination, lead to a profound inhibitory effect on pro-inflammatory cytokine production (129). On neonatal APCs, PTX demonstrates anti-inflammatory properties by (1) down-regulating TLR4 expression and signaling, (2) downregulation of surface molecules such as CD14 and CD11b, and (3) inhibition of inflammatory cytokine production (70). **(B)** The microbiome of premature infants has a smaller proportion of beneficial bacteria and higher numbers of pathogenic bacteria compared to term infants, likely owing to higher frequencies of cesarean sections, antibiotic use, exposure to the hospital environment, and artificial feeding (130). The administration of probiotics up-regulates local and systemic immunity by (1) decreasing proinflammatory cytokines, (2) increasing the production of anti-inflammatory cytokines, and (3) decreasing the permeability of the gut to bacteria and toxins (131). **(C)** Human milk contains a range of distinct bioactive molecules that protect against infection and inflammation including immunoglobulins, long-chain PUFAs, and LF. Among them, the antimicrobial and immunomodulatory effects of lactoferrin are best studied: (1) Inhibition of bacterial adhesion and biofilm formation (132–134), (2) binding of endotoxins from intestinal pathogens (135), (3) blocking of receptors essential for epithelial invasion of microbes (136) thereby (4) prevention of bacterial translocation (137), (5) promotion of anergic/anti-inflammatory effects in LPS or LTA stimulated macrophages by TLR expression and pathway interference (138, 139).

Human milk contains multiple distinct bioactive molecules, including antimicrobial proteins and peptides (APPs), that protect against infection and contribute to immune maturation, and healthy microbial colonization (Figure 1C) (140). Ethical limitations preclude RCTs on the topic but feeding preterm infants with their own mother's milk (MOM) offers an impressive array of benefits, including decreased rates of LOS, NEC and retinopathy of prematurity, lower rates of re-hospitalizations in the first year of life, and improved neurodevelopmental outcomes (141–144).

The term “human milk feeding” is frequently used to encompass both MOM and donor human milk (DHM), implying that the multiple beneficial outcomes attributed to MOM can be generalized to DHM (145, 146). This assumption, however, may only be partially correct due to differences in milk composition and processing. Preterm mothers' milk shows great variation in total protein levels and inverse correlation with lactation and daily milk volume (147). This demand-adapted regulation of protein intake, including APPs is hampered in DHM, because GA of donors' infants and infants receiving their milk might be mismatched or DHM might be pooled. More importantly, pasteurization of DHM destroys or significantly decreases the concentration of many of the protective elements in human milk, including lysozymes, secretory IgA, growth factors, lactoferrin, antioxidants, and microbiota (145, 146, 148–150). Nevertheless, improved outcomes of infants fed DHM may be primarily to avoiding potentially injurious effects of formula feeding (145, 146, 151). A meta-analysis showed that feeding with formula compared with DHM results in a higher risk of developing NEC (86). However, feeding DHM instead of formula did not significantly affect mortality or rate of invasive infection (Table 2) (86).

### Prebiotics

A wide range of prebiotic components (substrates that are selectively utilized by host microorganisms conferring a health benefit) and antimicrobial and anti-inflammatory factors are delivered by breast milk (141–143). These provide passive protection to the neonate and stimulate maturation of host intestinal defenses, which are particularly relevant for premature infants (144). Prebiotic components of human milk promote the growth of a physiologic, probiotic flora including *Bifidobacteria* spp. and *Lactobacilli* spp. in the colon. Although there is currently no evidence regarding the effectiveness of the isolated application of prebiotics in preventing nosocomial sepsis in preterm infants (152), combinations of probiotics and synbiotics, i.e., a synergistic combination of probiotics and prebiotics, may be beneficial for prevention of LOS, as described below (153).

### Probiotics

In healthy term infants the gut is colonized with maternal probiotic bacteria including *Lactobacilla* spp. and *Bifidobacteria* spp., which upregulate local and systemic immunity, increase anti-inflammatory cytokines, and decrease the permeability of the gut to bacteria and toxins (Figure 1B). Treatment with antibiotics and delayed enteral feeding are common in preterm infants and contribute to the development of sepsis and NEC (154). Probiotics, live non-pathogenic microorganisms, might confer a benefit to neonatal host immunity. By altering host epithelial and immunological responses (155), they may reduce several neonatal inflammatory conditions (156).

A 2014 Cochrane review of 24 randomized or quasi-RCTs evaluated the effect of probiotics in the prevention of NEC and LOS in preterm infants <37 weeks GA or <2,500 g birth weight, or both (157). Enteral probiotic supplementation significantly reduced the incidence of severe NEC ≥ stage II and mortality but initially, no evidence of significant reduction of LOS was found (157). Recent meta-analyses, including 37 RCTs (*n* = 9,416) have demonstrated a significant protective effect of probiotics against LOS and all cause mortality (87, 88, 158) (Table 2). The optimal composition of probiotics remains to be determined, but there is evidence that probiotics consisting of multiple strains are more effective than single-strain probiotics in preventing mortality and NEC (90).

Side effects of probiotic treatment are rare and most clinical studies did not report significant adverse effects. However, occasional cases of sepsis caused by administered probiotic species have been reported (159).

In summary, growing evidence suggests that the preventive use of probiotics reduces the risk for neonatal sepsis in preterm infants. Further studies are necessary to optimize formulation, composition, standardization and optimal dosing of probiotics.

### Synbiotics

The potential benefit of synbiotics, a combination of probiotics and prebiotics is currently being investigated and so far one large RCT has been published. This recent RCT of an oral synbiotics preparation (*Lactobacillus plantarum* plus fructo-oligosaccharide) enrolled 4,556 infants >2,000 g or 35 weeks of gestation, in rural India and found a significant reduction of the primary outcome sepsis and death in the treatment arm (risk ratio 0.60, 95% confidence interval 0.48–0.74) (160). Preterm infants represent a major challenge in resource-poor settings where NEC and sepsis carry greater risks of death (161). The risk–benefit ratio of prophylactic probiotics or synbiotics might differ between healthcare settings. Results indicate an equal or even more pronounced beneficial effect in neonates from developing countries and support further RCTs in both, high and low resource settings.

### Lactoferrin

Lactoferrin is the major whey protein in mature human milk and is present in even higher concentrations in colostrum (153). This multifunctional, 80 kDa iron-binding glycoprotein is part of the innate immune system and possesses a broad range of antimicrobial, immunostimulatory, anti-inflammatory, and anti-apoptotic properties (Figure 1C) (162).

Lactoferrin directly interacts with TLR4 and CD14 and demonstrates bacteriostatic activity through its high affinity for iron its ability to directly bind LPS (163, 164). In addition to its function as an antimicrobial protein, lactoferrin has also demonstrated immunoregulatory properties *in vitro* and *in vivo* (131, 165, 166). These studies indicate a potential role of lactoferrin as an immune-sensor to maintain immune homeostasis with an immunosuppressive effect on inflammatory monocytes/macrophages of preterm neonates (30, 131, 167).

Bovine lactoferrin (bLF) is only 69% homologous to human lactoferrin (hLF), but both serve similar biological functions (168). In RCTs the oral supplementation with bLF was associated with a decreased incidence of LOS caused by bacteria and invasive fungal infections in preterm infants (169). A recent Cochrane Review including six RCTs demonstrated that oral bLF supplementation with or without probiotics decreased LOS and NEC stage II or III but not mortality (Table 2) (75). Of note, to date no adverse effects regarding the use of bLF in human infants have been reported (75). Talactoferrin, a recombinant hLF, has been tested in a phase I study in preterm neonates (170). In contrast to bLF, talactoferrin does not need to be pasteurized and might therefore differ in function and effectiveness. Studies comparing talactoferrin and bLF remain to be done. Given the common use of probiotics, potential interactions between oral probiotics and bLF/talactoferrin should be tested (171).

Thus, optimum dosing regimens, type of lactoferrin (human or bovine), and effects on long-term outcomes still need to be defined, but lactoferrin might prove to be an effective agent in helping to prevent LOS in very preterm infants.

### Zinc

Preterm infants are born with low zinc (Zn) stores and a diminished capacity for Zn absorption and retention. Zn supplementation decreases oxidative stress markers and limits pro-inflammatory cytokine production by targeting Nuclear Factor Kappa B (NF-κB) (172, 173). A RCT in 352 infants aged 7–120 days with probable serious bacterial infection showed that Zn supplementation significantly reduced treatment failure, defined as need to change antibiotics, need for intensive care, or death (174). In a RCT high doses of Zn (9.7–10.7 mg/day) reduced mortality in preterm infants (24–32 weeks) without signs of infection at initial inclusion (175). Two RCTs have evaluated the effects of enteral Zn supplementation in preterm infants (≥32 weeks) with suspected sepsis (176, 177) and one of them (177) showed a significant reduction in mortality (178). A Cochrane review of the effects of enteral Zn supplementation in preterm neonates morbidity and mortality is currently pending (179).

### Pentoxifylline

Pentoxifylline (PTX), a non-specific phosphodiesterase inhibitor with immunomodulatory properties (Figure 1A), may be beneficial in preterm neonates with sepsis and NEC. PTX inhibits the production of TLR—and inflammasome-mediated inflammatory cytokines and the expression of LPS-stimulated surface markers *in vitro* (127, 128). PTX's effects are more pronounced in neonatal immune cells than in adults and it suppresses pro-inflammatory cytokines to a greater degree than anti-inflammatory cytokines (127, 128). This corresponds to the observation of limited clinical benefit in adult sepsis, but promising results from RCTs in neonates. Moreover, PTX's anti-inflammatory effects on PRR signaling are distinct from those of other anti-inflammatory agents such as dexamethasone and azithromycin with which PTX can act in synergy (180).

A meta-analysis of six RCTs encompassing 416 infants concluded that PTX, when used as an adjunct therapy to antibiotics in neonatal sepsis, might decrease mortality (Table 1) (77). Statistical subgroup analyses of four of these studies revealed lower mortality in preterm infants, infants with confirmed sepsis, and infants with Gram-negative sepsis. However, according to the authors, the overall quality of evidence was low (77). A recent double-blind RCT of 120 preterm infants with LOS demonstrated several beneficial adjuvant effects of PTX including a reduced length of hospital stay (*p* = 0.04), duration of respiratory support (*p* = 0.02) and less need for vasopressors (*p* = 0.01) but was not powered for clinical outcomes (181). Of note, no adverse effects of PTX were reported. Further *in vivo* studies, both in animal sepsis models and in human clinical trials, may help to define the optimal timing and dosing of PTX as well as its efficacy in improving short—and long-term outcomes following neonatal inflammation. Initiated in 2016, a randomized, placebo controlled multi-center study with a cohort size of 900 very preterm infants with LOS or NEC, is currently investigating PTX's effect on disability-free survival (Australian New Zealand Clinical Trials Registry 12616000405415).

## Future concepts with potential benefits

Although certain measures have indicated benefit, there remains an urgent and unmet need for novel, safe and efficacious strategies to reduce the huge burden of sepsis-related morbidity in the preterm population. We herein discuss new approaches with potential future applications that have not yet been tested beyond phase 1 clinical trials.

### Maternal immunization

Maternal immunization boosts the concentration of vaccine-specific IgG antibodies in the mother and increases antibody concentration in the infant at birth. Currently three vaccines have specific recommendations for routine use in pregnancy: tetanus, influenza and pertussis (182). Future prospects include potential development of maternal vaccines against GBS (183), cytomegalovirus (CMV) (184) and respiratory syncytial virus (RSV) (185).

In term infants, maternal vaccination may provide protection until the period of maximum susceptibility or risk has passed or until the infant has completed the routine immunizations. This benefit may be reduced in preterm infants due to reduced transplacental antibody transport before the third trimester with resulting lower antibody-concentrations compared to term infants (186). While much remains to be learned regarding the optimal timing, safety and efficacy of maternal vaccines as well as their potential effect on subsequent infant responses to vaccines (187), maternal immunization is an important strategy to substantially reduce morbidity and mortality from infectious diseases after birth.

### Antimicrobial proteins and peptides (APPs)

Both *in vitro* and *in vivo* data support the hypothesis that APPs are important contributors to intrinsic mucosal immunity. Alterations in the level of APP expression or biologic activity can predispose the organism to microbial infection (188). Primarily released by neutrophils, monocytes, and macrophages, APPs are also produced within the skin and at mucosal surfaces by epithelial cells and thus are present within body fluids, including saliva, airway surface liquid, and breast milk (189). Circulating and intracellular levels of APPs are relatively low in early life, especially in preterm infants, potentially lessening protective immunity (190). APPs expressed in neonates include α- and β-defensins, cathelicidins, bactericidal/permeability-increasing protein (BPI), and lactoferrin (191). While some studies argue for lactoferrin in the prevention of LOS in VLBW infants (192), preclinical data also support antibacterial and anti-endotoxin properties of therapeutic APPs. For example, the recombinant bactericidal/permeability-increasing protein (rBPI_21_) has demonstrated beneficial effects in children with severe meningococcal sepsis (193, 194). Further research on APPs for prevention and treatment of neonatal sepsis is ongoing and has recently been reviewed in detail (190). Synthetic peptides with combined antimicrobial and immunomodulatory properties, such as clavanin-MO, an adenosine monophosphate (AMP) isolated from *Styela clava*, and Innate defense regulator (IDR)-1018, derived from bovine bactenecin, represent a promising approach to treat invasive infections of various bacterial strains, including multidrug-resistant hospital isolates (195, 196). Of the APPs lactoferrin (Lf) has demonstrated benefit as oral agent in the prevention of neonatal sepsis (75). Regarding other APPs, although the results of preclinical studies are encouraging, to our knowledge neonatal clinical trials have not yet been conducted.

### Innate immune stimulants

Exposure to non-pathogenic components that augment the innate immune responses to prevent neonatal sepsis is promising, but has not yet been thoroughly investigated. Possible candidates are PRR- agonists; among them TLRAs are most extensively studied. In newborn mice, pre-treatment with TLRAs was associated with increased cytokine responses to subsequent polymicrobial infection, induced via intraperitoneal injection of a cecal slurry, with enhanced recruitment of phagocytes and reduced mortality (197). Although Th1-polarizing TLR- responses are diminished in preterm neonates compared to term neonates and adults (38, 39, 198), Th1 cytokine responses to TLR7/8 agonists such as R848 reach adult levels (37, 199). Whether any benefits confer to human neonates from TLRAs currently used as stand-alone agents (e.g., imiquimod cream, TLR7A) or as components of adjuvanted vaccines in clinical and preclinical trials in adults (200) needs to be carefully investigated in preclinical studies.

Most recently a beneficial effect of subcutaneously administered aluminum salts (alum) in the prevention of neonatal polymicrobial sepsis in mice was demonstrated (201). Alum, the most widely used adjuvant in human vaccines, does not activate TLRs but, rather, promotes caspase-1 activation and IL-1β production via the NACHT, LRR and PYD domains-containing protein 3 (NLRP3)-inflammasome (202).

Live attenuated vaccines such as Bacille Calmette–Guérin (BCG) provide inherent PRR-activating activity that may contribute to enhanced immune responses and a decreased susceptibility to invasive non-TB infections in developing countries, where BCG is routinely administered (54, 203). BCG may exert its beneficial potential through heterologous, “non-specific” effects. This heterologous innate immune protection may be due to “trained immunity,” the phenomenon whereby innate activation results in a heightened state of innate responses to a broad range of pathogens and thus broad protection, via innate memory as has been reviewed elsewhere (204). Of note, the combined administration of BCG plus the alum-adjuvanted hepatitis B vaccine (HBV) demonstrates age-dependent synergistic enhancement of IL-1ß, production potentially enhancing both innate and adaptive immune responses (205).

Thus, results from animal and human *in vitro* studies are indicative for a beneficial effect of innate immune stimulants in the prevention of neonatal sepsis and should be further investigated.

### Stem cells

Mesenchymal stromal cells (MSCs) are non-hematopoietic, multipotent stromal precursor cells that can be isolated from the placenta, cord blood, and Wharton's Jelly (206, 207). MSCs are capable of modulating immune responses (208) by both cell-to-cell contact and through the release of soluble paracrine factors including nitric oxide, indoleamine 2,3-dioxygenase, PGE_2_, TGF-β, and IL-10 (209, 210). MSCs may improve bacterial clearance by various mechanisms, including enhancement of phagocytic activity of APCs and up-regulation of TLR-2 and TLR-4, and β-defensin 2 secretion (211, 212). A comprehensive review of 18 preclinical studies published between 2009 and 2015 demonstrated that MSC therapy in animal models of sepsis significantly reduced the overall odds of death (OR 0.27, 95% CI 0.18–0.40) (213). A clinical trial of infusion of MSCs to adults demonstrated a significant increase in survival rate (214, 215). Further adult clinical trials are on-going, but to our knowledge neonatal sepsis trials have not yet been conducted (216). However, several studies currently investigate the use of umbilical cord blood derived-MSCs for the prevention and treatment of bronchopulmonary dysplasia (BPD) in human preterm infants at risk, after phase 1 trials showed an acceptable safety profile (217).

Of note, the abundance of hematopoietic stem and progenitor cells (HSPCs) in preterm compared to term cord blood, may contribute to the beneficial effects of delayed cord clamping in very preterm infants (218, 219). A recent systemic review of 2,834 preterm infants found high-quality evidence for reduced hospital mortality, but no clear evidence for a reduction of LOS in infants with delayed cord clamping (220).

### Inflammasome inhibitors

The inflammasome is a newly identified group of PRRs and specific blockage of these by small-molecule inflammasome inhibitors is a promising approach in different inflammatory conditions, including microbe-induced inflammation (221, 222). Numerous inflammasomes have been described so far; among them the (NLRP3) has been best characterized. Interactions among the three proteins of the NLRP3 inflammasome (NLRP3 protein, adapter protein apoptosis-associated speck-like protein (ASC) and procaspase-1) tightly regulate inflammasome function to ensure immune activity only when appropriate. *In vivo* neutralization of the NLRP3 inflammasome with an orally available small molecule inhibitor decreased inflammasome dependent cytokine secretion (IL-1β /IL-18) in a murine *Staph. aureus* infection, and improved the bacterial clearance through improved acidification of the phagosome (223). Newborn neonatal caspase-1/11 knockout mice, showed improved survival following septic challenge compared with wild-type mice (224). This effect was independent of NLRP3 activation (224). Despite promising results from clinical trials in adults treated with inflammasome inhibitors for inflammatory diseases other than sepsis (225, 226), more information is needed regarding the mode of inflammasome action in neonates to inform potential targeted therapeutic inhibition in this distinct age group.

### Antibiotics with anti-inflammatory properties

Inflammation in sepsis is usually triggered by microbial components, hypoxia, arterial hypotension, and reperfusion. Some antimicrobial agents, such as β-lactam antibiotics, may exacerbate inflammation through the lysis of bacteria (227).

In contrast to proinflammatory effects of certain antibiotics that lyse bacteria, several protein synthesis inhibiting antibiotics exhibit anti-inflammatory activities. Macrolides, rifampicin, and tetracycline demonstrate anti-inflammatory and immunomodulatory properties with a potential application in systemic inflammation (228–231). Rifampicin and tetracycline are contraindicated in the neonatal period; the use of macrolides for other indications, namely, *Ureaplasma spp*. infection and the prevention of BPD in preterm neonates, however, has been studied (232, 233). Prophylactic azithromycin significantly reduced BPD and the composite outcome of BPD/death in preterm infants (232).

The macrolide azithromycin, specifically inhibits IL-1α and IL-1β secretion and non-canonical inflammasome activation upon TLR agonist, including LPS, stimulation *in vitro* (180) and *in vivo* (234). A decrease in IL-1β-mediated inflammation has been attributed to destabilizing mRNA levels for NALP3, a key inflammasome component (235). In a murine sepsis model, mortality was lower along with decreased sepsis scores when mice were treated with a combination of ampicillin and azithromycin instead of ampicillin alone (236). In adults, azithromycin was associated with more ICU-free days in severe sepsis patients with and without pneumonia (237), however questions on the applicability of these results in neonates remain including the consideration of side effects (238–240).

None of these new approaches has been tested in human neonates thus far and the potential *in vitro* and *in vivo* effects of new therapies need to be fully explored before any clinical studies in neonates can be performed. In addition to possibly beneficial single agent such as PTX, future studies will evaluate if a multimodal approach including a combination of immunomodulatory agents may prevent or mitigate neonatal sepsis and associated long term- morbidities in preterm infants.

## Conclusion

Administration of human milk is a key approach in preventing neonatal sepsis. The use of probiotics and lactoferrin might be effective but more evidence is needed to confirm preliminary observations and to optimize formulation, composition, and dosing of these agents. In certain settings/populations, vaccination with BCG is associated with a reduction in neonatal sepsis via heterologous (“non-specific”) effects possibly related to trained immunity. With respect to treatment of neonatal sepsis, PTX holds promise, but larger studies with long-term outcome data are still pending. Several other immunotherapies evaluated for the prophylaxis of neonatal sepsis including IVIG, myeloid CSFs and granulocyte transfusions have failed to demonstrate benefit. As we look to the future, APPs, PRR-agonists, stem cell therapy and inhibitors of the inflammasome might offer new therapeutic or preventive avenues in neonatal sepsis with preclinical and clinical studies yet to be done.

The successful development of new prophylactic and treatment options should take into account age-specific immune responses. Timing of therapy and dosage may determine whether immunomodulatory agents induce a protective immune response or whether the same approach causes potentially harmful interference with the developing immune system.

## Author contributions

SS conducted the literature review and drafted the paper. EV designed the tables and assisted in drafting. BK, AS, OL, and AB contributed to critical revision of the article, assisted in drafting, and suggested additional references for inclusion in the final version. OL edited the final manuscript. All authors approved the final manuscript as submitted.

### Conflict of interest statement

In addition to its major funding via the National Institutes of Health, the Levy Lab has periodically received sponsored research support from companies that develop anti-infectives, adjuvants and/or adjuvanted vaccines such as Johnson & Johnson (Janssen Pharmaceuticals), MedImmune, Shire, and 3M Drug Delivery Systems. OL is an inventor on a licensed patent on use of the antimicrobial protein rBPI21 to mitigate radiation injury and on several patent applications regarding vaccine adjuvants. The remaining authors declare that the research was conducted in the absence of any commercial or financial relationships that could be construed as a potential conflict of interest.
