# Supplementary material for: Immunomodulation to Prevent or Treat Neonatal Sepsis: Past, Present, and Future
Source: Front Pediatr. 2018 Jul 19;6:199. doi: 10.3389/fped.2018.00199 (PMC6060673; doi:10.3389/fped.2018.00199)
Supplement: Supplementary file 1 [file Data_Sheet_1.docx]

Supplementary Material

**Immunomodulation to Prevent or Treat Neonatal Sepsis: Past, Present, and Future**

Simone S. Schüller*, Boris W. Kramer, Eduardo Villamor, Andreas Spittler, Angelika Berger and Ofer Levy*

*** Correspondence:**

Simone S. Schüller: [simone.schueller@childrens.harvard.edu](mailto:simone.schueller@childrens.harvard.edu)

Ofer Levy: [ofer.levy@childrens.harvard.edu](mailto:ofer.levy@childrens.harvard.edu)


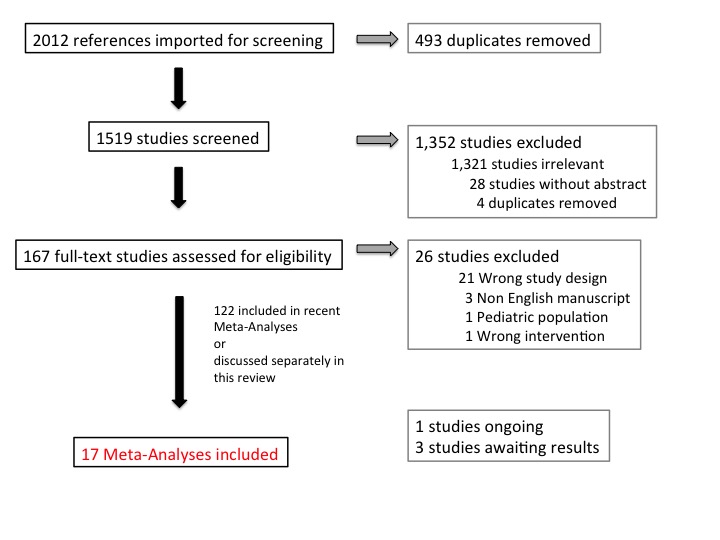


**Supplementary Figure 1. Literature search strategy** A literature search in Pubmed, Embase, Web of Science, NIH US National Library of Medicine (ClinicalTrials.gov) and the Australian New Zealand Clinical Trials Registry (ANZCTR; anzctr.org.au) was performed (-March 2018)

The following search terms were used for the literature search in Supplementary Figure 1:

**CONCEPT 1:**

PUBMED

("Neonatal Sepsis"[Mesh] OR (neonatal[tiab] AND sepsis[tiab]) OR (neonatal[tiab] AND septicaemia[tiab]) OR (neonatal[tiab] AND septicemia[tiab]) OR (newborn[tiab] AND septicaemia[tiab]) OR (newborn[tiab] AND septicemia[tiab]))

Embase

('newborn sepsis'/exp OR (neonatal:ab,ti AND sepsis:ab,ti) OR (neonatal:ab,ti AND septicaemia:ab,ti) OR (neonatal:ab,ti AND septicemia:ab,ti) OR (newborn:ab,ti AND septicaemia:ab,ti) OR (newborn:ab,ti AND septicemia:ab,ti))

**CONCEPT 2:**

PUBMED

("Chemotherapy, Adjuvant"[Mesh] OR ((Adjunctive[tiab] OR Adjuvant[tiab]) AND (therapy[tw] OR treatment[tw])) OR "Antimicrobial Peptides"[tiab] OR "Antimicrobial Proteins"[tiab] OR "Antimicrobial Cationic Peptides"[Mesh] OR "Antimicrobial Cationic Peptides"[tiab] OR "Antimicrobial Cationic Peptide"[tiab] OR "Lactoferrin"[Mesh] OR Lactoferrin[tiab] OR Glutamine[tiab] OR Glutamin[tiab] OR "Selenium"[Mesh] OR Selenium[tiab] OR drotrecogin[tw] OR retinol[tiab] OR "Vitamin A"[Mesh] OR "Vitamin A"[tiab] OR "Vitamin E"[tiab] OR TOCOPHEROL*[tiab] OR "Blood Transfusion"[tiab] OR "Blood Transfusion"[Mesh] OR "Exchange transfusion"[tiab] OR "Exchange Transfusion, Whole Blood"[Mesh] OR Melatonin[Mesh] OR Melatonin[tiab] OR "Activated human protein C"[tiab] OR "Milk, Human"[Mesh] OR "human milk"[tiab] OR "breast milk"[tiab] OR Probiotics[Mesh] OR "Synbiotics"[Mesh] OR Synbiotic*[Mesh] OR "Prebiotics"[Mesh] OR Prebiotic*[tiab] OR "altastaph"[Supplementary Concept] OR Altastaph[tiab] OR "pagibaximab"[Supplementary Concept] OR BSYX-A110[tiab] OR "Veronate"[Supplementary Concept] OR Veronate*[tiab] OR "INH-A00021"[tiab] OR INH-A21[tiab] OR "Granulocyte Colony-Stimulating Factor"[Mesh] OR GM-CSF[tiab] OR "Granulocyte transfusion"[tiab] OR G-CSF[tiab] OR "Granulocyte-Macrophage Colony-Stimulating Factor"[Mesh] OR "Granulocyte Macrophage Colony Stimulating Factor"[tiab] OR "CSF-2"[tiab] OR "GM Colony-Stimulating Factor"[tiab] OR "GM-CSF"[tiab] OR CSF-GM[tiab] OR "Immunoglobulins, Intravenous"[Mesh] OR immunoglobulins[tiab] OR "Pentoxifylline"[Mesh] OR "Pentoxifylline"[tiab])

EMABASE

('adjuvant therapy'/exp OR ((Adjunctive:ab,ti OR Adjuvant:ab,ti) AND (therapy:ab,ti OR treatment:ab,ti)) OR "Antimicrobial Peptides":ab,ti OR "Antimicrobial Proteins":ab,ti OR 'antimicrobial cationic peptide'/exp OR 'antimicrobial cationic peptide':ab,ti OR 'antimicrobial cationic peptides':ab,ti OR 'lactoferrin'/exp OR 'lactoferrin':ab,ti OR "lactoferrine":ab,ti OR lactotransferrin:ab,ti OR 'talactoferrin'/exp OR 'talactoferrin':ab,ti OR 'glutamine'/exp OR Glutamine:ab,ti OR Glutamin:ab,ti OR 'selenium'/exp OR “Selenium":ab,ti OR 'retinol'/exp OR “vitamin A”:ab,ti OR 'tocopherol'/exp OR "Vitamin E":ab,ti OR TOCOPHEROL*:ab,ti OR "Blood Transfusion":ab,ti OR 'blood transfusion'/exp OR “blood exchange”:ab,ti OR “blood infusion”:ab,ti OR "Exchange transfusion":ab,ti OR 'melatonin'/exp OR Melatonin:ab,ti OR "Activated human protein C":ab,ti OR 'drotrecogin'/exp OR 'drotrecogin':ab,ti OR 'breast milk'/exp OR 'breast milk':ab,ti OR "human milk":ab,ti OR 'probiotic agent'/exp OR 'probiotic agent':ab,ti OR 'probiotic agents':ab,ti OR 'synbiotic agent'/exp OR Synbiotic*:ab,ti OR 'prebiotic agent'/exp OR Prebiotic*:ab,ti OR "altastaph":ab,ti OR 'pagibaximab'/exp OR pagibaximab:ab,ti OR BSYX-A110:ab,ti OR Veronate*:ab,ti OR "INH-A00021":ab,ti OR INH-A21:ab,ti OR 'recombinant granulocyte colony stimulating factor'/exp OR 'granulocyte colony stimulating factor'/exp OR "Granulocyte Colony-Stimulating Factor":ab,ti OR GM-CSF:ab,ti OR "Granulocyte transfusion":ab,ti OR G-CSF:ab,ti OR 'granulocyte macrophage colony stimulating factor'/exp OR 'recombinant granulocyte macrophage colony stimulating factor'/exp OR "Granulocyte Macrophage Colony Stimulating Factor":ab,ti OR "CSF-2":ab,ti OR "GM Colony-Stimulating Factor":ab,ti OR "GM-CSF":ab,ti OR CSF-GM:ab,ti OR 'immunoglobulin'/exp OR immunoglobulins:ab,ti OR 'pentoxifylline'/exp OR "Pentoxifylline":ab,ti)

RCT Filter PubMed

"randomized controlled trial"[pt] OR "controlled clinical trial"[pt] OR randomized[tiab] OR placebo[tiab] OR "drug therapy"[sh] OR randomly[tiab] OR trial[tiab] OR groups[tiab]

RCT Filter EMBASE

'randomized controlled trial'/exp OR 'controlled clinical trial'/exp OR randomized:ti,ab OR placebo:ti,ab OR 'drug therapy':lnk OR randomly:ti,ab OR trial:ti,ab OR groups:ti,ab

For the search of ongoing clinical trials NIH US National Library of Medicine (ClinicalTrials.gov) and the Australian New Zealand Clinical Trials Registry (ANZCTR; anzctr.org.au) we used the concept “neonatal sepsis”.

All findings were uploaded on a cloud-based software (<https://www.covidence.org>) and screened according to title/ abstract and full text (see workflow in Supplementary Figure 1).
